# A single-blind, pilot randomised trial of a weight management intervention for adults with intellectual disabilities and obesity: study protocol

**DOI:** 10.1186/2055-5784-1-5

**Published:** 2015-01-12

**Authors:** Leanne Harris, Craig Melville, Nathalie Jones, Carol Pert, Susan Boyle, Heather Murray, Janet Tobin, Fiona Gray, Catherine Hankey

**Affiliations:** 1 College of Medical Veterinary and Life Sciences, Institute of Mental Health & Wellbeing, University of Glasgow, Glasgow, G12 0XH UK; 2 Learning Disabilities Psychiatry, Institute of Health & Wellbeing, College of Medical Veterinary and Life Sciences, University of Glasgow, Academic Unit for Mental Health & Wellbeing, Gartnavel Royal Hospital, 1st floor Administrative Building, 1055 Great Western Road, Glasgow, G12 0XH UK; 3 Learning Disability Psychology NHS Greater Glasgow and Clyde, Glasgow, G52 2HH UK; 4 Glasgow and Clyde Weight Management Service, Glasgow, G41 3DX UK; 5 Robertson Centre for Biostatistics, University of Glasgow, Glasgow, G12 8QQ UK; 6 Glasgow City CHP North East Sector, Eastbank Conference Training Centre, Glasgow, G32 9AA UK; 7 North East Quadrant Shettleston Health Centre, Glasgow, G32 7JZ UK; 8 Human Nutrition, College of Medical Veterinary and Life Sciences, University of Glasgow, Glasgow, G31 2ER UK

**Keywords:** Intellectual disability, Weight management, Obesity, TAKE 5

## Abstract

**Background:**

The prevalence of obesity in adults with intellectual disabilities has consistently been reported to be higher than the general population. Despite the negative impact of obesity on health, there is little evidence of the effectiveness of weight management interventions for adults with intellectual disabilities and obesity. Preliminary results from a single-stranded feasibility study of a multi-component weight management intervention specifically designed for adults with intellectual disabilities and obesity (TAKE 5) and that satisfied clinical recommendations reported that it was acceptable to adults with intellectual disabilities and their carers. This study aims to determine the feasibility of a full-scale clinical trial of TAKE 5.

**Methods:**

This study will follow a pilot randomised trial design. Sixty-six obese participants (body mass index (BMI) ≥30 kg/m^2^) will be randomly allocated to the TAKE 5 multi-component weight management intervention or a health education ‘active’ control intervention (Waist Winners Too (WWToo)). Both interventions will be delivered over a 12-month period. Participants’ anthropometric measures (body weight, BMI, waist circumference, percentage body fat); indicators of activity (levels of physical activity and sedentary behaviour) and well-being will be measured at three time points: baseline, 6 and 12 months. The researcher collecting outcome measures will be blind to study group allocation.

**Conclusions:**

The data from this study will generate pilot data on the acceptability of randomisation, attrition rates and the estimates of patient-centred outcomes of TAKE 5, which will help inform future research and the development of a full-scale randomised clinical trial.

**Trial registration:**

ISRCTN52903778.

## Background

Intellectual disability is defined as the ‘disability characterised by significant limitations both in intellectual functioning and in adaptive behaviour, which covers everyday social and practical skills. The disability originates before the age of 18 years [1]’. Individuals with intellectual disabilities have consistently been reported to have higher rates of obesity than the general population [2–6]. Obesity, defined as a body mass index (BMI) of 30 kg/m^2^ or greater, is known to have a negative impact on health, associated with chronic diseases such as cardiovascular disease [7], some cancers [8] and type II diabetes [9]. Being overweight or obese has shown to further exacerbate the health needs and already reduced life expectancy of adults with intellectual disabilities [10]. Despite the negative impact of obesity on health, there is a limited evidence base to inform the management of obesity in this population [11].

Clinical guidance on the management of obesity advocates that adults who are overweight or obese should aim for a clinically important, sustainable weight loss of 5%–10% of initial body weight [12, 13]. To achieve this multi-component weight management, interventions are recommended which include as follows:

1. Dietary changes to create an energy deficit diet (EDD) of 2,510 kilojoules (kJ)/day (600 kilocalories (kcal/day).
2. Support to increase physical activity levels and decrease inactivity.
3. Incorporation of behaviour change strategies to facilitate dietary and activity changes.
4. A weight maintenance phase encouraging sustained behaviour changes in healthy eating, increased physical activity and reduced sedentary behaviour.
5. A minimum 12-month study period (including the intervention and follow up) to examine the efficacy of the intervention.

Multi-component interventions have been used in the general population to successfully support individuals to lose a clinically important weight [14]. However, there are no published randomised controlled trials of weight management interventions meeting clinical recommendations in adults with intellectual disabilities.

Prior to this study, a single-stranded feasibility study was carried out to examine the efficacy of TAKE 5; a multi-component weight management intervention specifically designed for adults with intellectual disabilities and obesity [15, 16]. TAKE 5 was developed in collaboration with National Health Service (NHS) Greater Glasgow & Clyde Weight Management Service (GCWMS) [14] and modelled on their multi-component intervention, which incorporates diet and activity advice underpinned by behaviour change approaches and based on clinical guidelines [12, 13]. Full results from the TAKE 5 feasibility study have been published previously [15, 16]. The feasibility study found that TAKE 5 was acceptable to adults with intellectual disabilities, and carers, and reported clinically important reductions in body weight which were comparable to those achieved in adults with no reported intellectual disability following the GCWMS multi-component intervention [17]. Furthermore, clinically important reductions in risk factors associated with chronic diseases, such as waist circumference and increased physical activity levels, were also observed [15, 16, 18].

This study will add to the limited evidence base of controlled trials which have examined the efficacy of weight management interventions for adults with intellectual disabilities by piloting an intervention meeting current UK clinical recommendations on weight management. This study will compare the effects of a multi-component weight management intervention, TAKE 5 with a health education control intervention which does not include quantitative dietary advice to generate an individualised energy deficit, Waist Winners Too (WWToo). The design and rationale of the study with detail on the components of both interventions will be reported in this protocol.

### Aim

The overall aim of this pilot randomised trial is to examine the feasibility of a full-scale clinical trial of the TAKE 5 multi-component weight management intervention in comparison with a health education control intervention.

The key research questions to determine the feasibility of a full-scale randomised clinical trial are the following:

1. Can adults with intellectual disabilities and obesity be recruited to a randomised study of the TAKE 5 intervention versus a health education control intervention?
2. What attrition rates are observed at 6 and 12 months post-randomisation?
3. Are the patient centred outcome measures acceptable to the participants and can they be measured reliably to detect clinically important changes?
4. What are the sample size requirements for a full-scale clinical trial powered at 90% to determine a clinically important difference in body weight?

## Methods

The study will be conducted in accordance with the ethical principles of the Declaration of Helsinki and consistent with the principles of Good Clinical Practice. Ethical approval has been received from the Scotland A Research Ethics committee. In accordance with the Adults with Incapacity (Scotland) Act 2000, a detailed protocol of consent was implemented. This included seeking consent from individuals with intellectual disabilities with the capacity to provide informed consent and seeking consent from the nearest relative or welfare guardian in circumstances where the individual was unable to provide informed consent. Written informed consent was obtained from all participants or nearest relative or welfare guardian. Participants will have the right to withdraw from the study at any time and to decline to take part in any particular aspect or measure. This will be explained to them whilst seeking consent and their on-going consent will be checked and assessed throughout the intervention.

### Design

**Figure 1:**
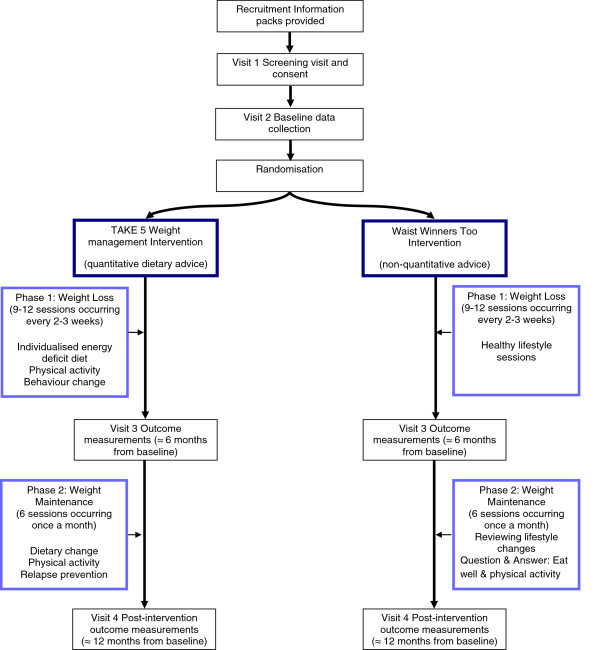
**Study flow chart.**

This study is a single-centre, single-blind pilot randomised trial. It consists of two active intervention arms: TAKE 5 multi-component weight management intervention versus treatment as usual (TAU), health educational intervention, WWToo. TAU in most areas in the UK for adults with intellectual disabilities and obesity is inconsistent—ranging from no intervention to the WWToo health education approach. An ‘active’ control intervention is preferred to the traditional non-intervention control group as due to the health risks associated with obesity [19, 20]; the research group believe it to be unethical to offer participants to be randomised to the control group with no intervention, for a 12-month period. Sixty-six participants will be randomised to the study (33 to each treatment arm) for a 12-month period; a 6-month weight loss period (comprising of 9–12 sessions designed to take place between two and three weekly intervals) followed by a 6-month weight maintenance period (comprising of six sessions taking place once a month). If the participants have not lost a clinically relevant weight loss of 5% of initial body weight at the end of the weight loss period, they will be advised to continue on the weight loss plan for a further 3 months, followed by a condensed 3-month weight maintenance period, mirroring the procedures conducted by the GCWMS and allowing a 12-month ‘intervention period’ for all participants. The study design is illustrated in Figure 1.

### Study population

Participants will be invited to take part in the study if they meet the eligibility criteria presented in Table 1.

**Table 1:** **Participant eligibility, inclusion and exclusion criteria**

Criteria	
Inclusion	
Intellectual disability	Any level of intellectual disability
Age	Adults ≥18 years old (there is no upper age limit, in keeping with the GCWMS and specialist intellectual disability health services in NHS GGC)
Weight status	BMI ≥30 kg/m^2^
Ambulatory	Ability to walk (with or without a walking aid) for 10 min at a time based on self/carer report
Diet	Not currently on a prescribed or restricted diet, e.g. for phenylketonuria or diabetes
Weight stability	No intentional weight loss >3 kg over the previous 3 months
Exclusion	
Genetic intellectual disability	Participants with Prader Willi syndrome, Cohen syndrome or Bardet-Biedl syndrome as they require specific support to lose weight
Research	Currently taking part in any other research study
Medication	Taking medication; either prescribed or over the counter, designed for weight loss
Pregnancy	Individuals who are pregnant will be excluded from the study and anyone who conceived during the study will be excluded.

### Recruitment

One aim of the proposed study is to examine recruitment and retention to a randomised trial design and inform recruitment strategies that could be used in the development of a full-scale, multi-centre trial. Therefore, this pilot randomised trial will use a multi-point recruitment strategy, involving primary health care services, specialist intellectual disability services, relevant voluntary organisations and care provider organisations. A record will be kept of the numbers of potential participants, and individuals consenting to participate in the study, identified from each of the recruitment points.

The research team will visit staff working in these settings to explain the study and ask if their organisation would be willing to support recruitment to the study. For those staff willing to support recruitment to the study, a supply of study information packs will be provided, comprising information about the study including an invitation for potential participants to participate. Staff will be invited to distribute these packs to service users who they think would fulfil the study inclusion criteria and potentially be willing to take part. Potential participants, who reply to the invitation to the study by post using the self-addressed envelope provided, will indicate whether they would like to meet the researcher to find out more about the study. The researcher (LH) will then contact participants to arrange a visit, at a convenient location to the participant, to discuss the study. Individuals recruited to the study may live together and/or be supported by the same family or paid carers. These factors could make it difficult to randomise people to the different treatment interventions of the study and lead to some contamination between treatments and clustering of outcomes. Cluster randomisation, stratified by (and therefore analysed with adjustment for) the number of individuals within a randomised cluster, level of intellectual disabilities and presence of Down Syndrome will account for the clustering of outcomes and minimise imbalance between study groups.

### Sample size

There is limited data from controlled trials of weight management involving adults with intellectual disabilities on which to base a sample size calculation. This study is designed to estimate recruitment and retention rates for a full-scale clinical trial; it is not powered to detect a difference between study groups. Sixty-six participants will be recruited (33 to each treatment arm). This will provide sufficient insight into recruitment and retention rates, which will have a 95% confidence interval of no more than ±10%. The sample size will allow for a possible attrition rate of 20%. This study also aims to determine likely variance of study outcomes in order to power a larger randomised trial; if 50 participants provide outcome data at 12 months, a 90% confidence interval for each variance estimate will have a width of approximately 70% of the estimate (i.e. −26% to +44%).

### Procedure

Participant eligibility will be assessed by the researcher based on the inclusion and exclusion criteria (Table 1) at an initial screening visit. Participants will also be asked to complete the Physical Activity Readiness Questionnaire (PARQ) [21] in order to identify any potential contraindications to increasing their physical activity levels. If participants score positively, they will be advised to consult their doctor about whether they should participate in the study.

After obtaining informed consent, the researcher will arrange a visit to collect baseline outcome measures. Participants’ level of intellectual disability will be assessed with questions assessing an individual’s level of ability and need for support in five key areas of functioning: eating and drinking, intimate care, personal safety, communication and decision making. A total score (range 5–25) is obtained by adding together the scores from the five individual questions. This will be used to categorise the level of intellectual disability as mild, moderate, severe or profound. The cut-offs corresponding to the four categories of intellectual disabilities were derived in a previous study [22] and shown to have a good level of agreement with a validated structured assessment of functioning and ability level, the Vineland’s Adaptive Behaviour Scale [23]. Participants will then be randomised into the TAKE 5 intervention or WWToo intervention. The researcher will telephone an interactive voice response system (IVRS), hosted by the Robertson Centre for Biostatistics, University of Glasgow. The researcher will register each participant in the study, by giving the participants’ cluster number, the number of individuals within the cluster, level of intellectual disabilities and presence of Down Syndrome. After registering each participant, the system will notify the principal investigator of the allocation (TAKE 5 intervention or WWToo intervention). During post randomisation, participants will be contacted by the research dietitian delivering the interventions and arrangements will be made for their first session. Intervention sessions will take place in a participant’s own home or, if an individual prefers, in out-patient facilities located in the local learning disabilities team base, or other NHS Greater Glasgow & Clyde setting. The researcher collecting the data will be blind to group allocation. After the first 6 months of weight loss phase and at 12 months, all outcome measures will be repeated. These data will be used to determine whether any changes in outcome measures have been maintained.

### Interventions

Both interventions will be delivered by a research dietitian. The session frequency (9–12 sessions in the weight loss phase and six sessions in the weight maintenance phase) is to allow appointments to be organised flexibly to maximise the consistent involvement of family and paid carers. Previous research has shown that the involvement of family and paid carers contributes to the effectiveness of weight management interventions for adults with intellectual disabilities [24, 25]. Each session will last approximately 40–60 min duration. This is to allow some flexibility in the session content, required to take account of the individual needs and abilities of participants. For example, some participants and carers may prefer to have shorter sessions or have extra sessions to develop an understanding of the information. This study will use appropriate methods and techniques for augmentative communication, e.g. talking mats and pictorial explanations that aim to enable participants to express their choices during the intervention [26]. Accessible resources, appropriate to the developmental levels of adults with intellectual disabilities were developed during the TAKE 5 feasibility study and will be used to support participants in both intervention arms. Supporting resources are designed to be used flexibly with adults with all levels of learning disabilities, involving family and paid carers where appropriate.

#### TAKE 5

TAKE 5 is an individualised intervention involving family or paid carers to support participants, where appropriate. It was adapted specifically for use with adults with intellectual disabilities in that it is designed to be delivered on a one-to-one basis with support from carers in the individual’s home environment instead of a group setting as in the GCWMS intervention [13].

#### TAKE 5 intervention components

The main themes discussed at each session for weight loss and weight maintenance are illustrated in Tables 2 and 3, respectively. Each session will focus on a discussion point on diet and physical activity and incorporate behavioural change techniques (Table 2).

**Table 2:** **Intervention key themes underpinning weight loss sessions**

Session	TAKE 5	Waist Winners Too
1	Benefits of losing weight and motivation towards a healthy lifestyle	Introduction to health and weight
2	Introduction to individualised energy deficit diet and the importance of physical activity	Planning meals
3	Principles of healthy eating and improving physical activity levels	Food labelling and fats
	Introduction to physical activity diaries and pedometer	
4	Healthy ways to cook, meal planning and shopping lists	Food labelling salt and sugar
	Emotions and overeating	
5	Disadvantages of eating out and take-aways	Shopping, budgeting, snacks, eating out and take-aways
	Using behaviour change to alter ‘bad habits’	
6	Coping with cravings and evaluating knowledge of physical activity	Alcohol and other drinks
7	Diet myths and introduction to new ways to motivate participation in physical activity	Benefits of exercise
8	Relapse prevention	Review
9	Evaluate success up to now	What have we learned

**Table 3:** **TAKE 5 key themes underpinning weight maintenance sessions**

Session	Theme
1	Weight maintenance and new individualised maintenance dietary plan
2	Importance of being active and adopting regular eating patterns
3	Regular self-monitoring of weight and food intakes
4	Overview of barriers to healthy eating and physical activity
5	Snacking, lapses, eating out/social activities
6	Healthy menu plan and review of principles of weight maintenance

### Diet

#### Phase 1—weight loss

To achieve a healthy sustainable weight loss of 0.5–1.0 kg per week, a daily EDD of 600 kcal is recommended [11, 12]. Each individual’s daily energy deficit will be calculated based on the estimate of their total energy expenditure −2,510 kJ (600 kcal). Basal metabolic rate (BMR) will be calculated with gender, age, height and weight using the Mifflin St. Jeor equation [27]. Total energy expenditure is estimated from BMR multiplied by a physical activity level of 1.3 [28].

The EDD provides daily caloric intake from a specified number of daily portions from the five food groups in the *Eatwell* plate: starchy foods such as bread, rice, potatoes and pasta; meat/fish and alternatives; fruit and vegetables; milk and dairy products; foods high in sugar and fat [29]. The EDD also gives specific advice on portion sizes and alternatives to energy dense food stuffs.

#### Phase 2—weight maintenance

In the weight maintenance phase, dietary intake will continue to be based on the principles of portions from the five food groups from the *Eatwell* plate [29]. However, this will not incorporate a daily EDD approach to include a deduction of 600 kcal. Instead, each individual’s energy requirements will be calculated to maintain energy balance.

### Physical activity

#### Phase 1—weight loss

Results from the single-stranded feasibility study of TAKE 5 reported that individuals with intellectual disabilities and obesity have very sedentary lifestyles and have low levels of physical activity spending an average of 13.1 (SD 16.2) min per day in moderate-to-vigorous-intensity physical activity [15]. The majority of physical activity recommendations advocate 30 min of moderate-to-vigorous physical activity on most days of the week [30, 31]. This may be unrealistic and unattainable for some individuals with intellectual disabilities. Therefore, consensus guidelines on physical interventions for beginners [32] will be adhered to, initially aiming to support participants to progressively increase regular participation in physical activity and reduce time spent being sedentary.

In each session, physical activity goals will be negotiated and set based on the individual’s current level of physical activity, physical ability and individual’s expressed preference, with the overall aim to gradually work towards current physical activity recommendations advocated for all adults.

Participants will be encouraged to reduce the time spent sedentary, i.e. watching TV, by accumulating bouts of physical activity over the course of the day. Current activity across three types of physical activity will be reviewed with each participant and carer:

- Activity at home as a replacement for sedentary behaviour, e.g. housework and gardening
- Walking—based on baseline average steps per day, individuals will be encouraged to set targets to progressively increase walking behaviour and use pedometers to monitor step counts
- Sport and exercise—information will be given to each participant on local leisure facilities and clubs with disability accessible sports and exercise groups/classes.

#### Phase 2—weight maintenance

The importance of physical activity will be highlighted in the maintenance phase as it plays an important role in sustaining any reductions in body weight [12, 13]. Individuals will be encouraged to build on the levels of physical activity they achieved in phase 1 and continue to aim to meet clinical recommendations.

### Behaviour change techniques

#### Phase 1—weight loss

It is recommended that behaviour change techniques are incorporated into weight management interventions to support and maintain changes in attitudes and behaviour in relation to healthy lifestyle patterns such as healthy eating, increased physical activity and a decrease in sedentary behaviour [12, 13]. The techniques incorporated into the TAKE 5 intervention are based on the recommendation of clinical guidelines and will include self-monitoring, goal setting, management of eating behaviours, relapse prevention, stimulus control, cue avoidance, reinforcement and social assertion [15, 18]. There are recognised challenges to supporting behaviour change in adults with intellectual disabilities. Therefore, TAKE 5 uses support from family or paid carers to provide encouragement and motivation for behaviour change and the behavioural methods above flexibly in keeping with the needs and ability of levels of participants [33].

Goal setting and dietary self-monitoring are key aspects of behaviour change [13]. Short-term goals incorporating dietary change and physical activity will be set by participants at the end of each session and reviewed subsequently. Participants and carers will be provided with daily diaries to facilitate monitoring of dietary intakes and level of participation in physical activity. These will be reviewed at each session and used to monitor progress towards goals, identify potential barriers to change and discuss means to achieve goals. This information is used as part of the intervention and will not be used as a source of data for formal statistical analyses. When an individual’s level of abilities allows additional behavioural change, techniques will be included within the TAKE 5 intervention, such as problem solving and assertiveness.

#### Phase 2—weight maintenance

To maintain a healthy body weight, behavioural strategies used in the weight loss phase will continue to be used flexibly. Specific approaches, such as problem solving and lapse and relapse prevention, are particularly relevant to discuss and use during maintenance sessions. Participants will be encouraged with support from carers where appropriate to maintain the healthy lifestyle habits from phase 1. Furthermore, they will be encouraged to regularly self-monitor their body weight, food intake and habitual physical activity. Goal setting will be used at the end of each session (Table 3).

#### Control intervention (WWToo)

WWToo is a health education intervention developed by a partnership group consisting of NHS dietitians, intellectual disability nurses, health improvement specialists and representatives from Glasgow Life, responsible for delivering community leisure facilities. WWToo was adapted from a mainstream Waistwinners programme into an accessible format for adults with intellectual disabilities. The Waistwinners programme aims to increase knowledge and skills to improve behaviour related lifestyle habits such as healthy eating, weight management and physical activity. The programme is a group-based intervention delivered over eight weekly sessions.

For the purpose of this research study, the delivery of the WWToo intervention has been modified to an intervention delivered on a one-to-one basis. Participants in this control intervention will receive the same number of face-to face sessions as participants in the TAKE 5 intervention. To retain participants to the study for the same duration as TAKE 5, a weight maintenance phase to WWToo was developed.

### WWToo intervention components

#### Phase 1—weight loss

##### Diet

Dietary change will be based on non-quantitative advice based on the food groups from the *Eatwell* plate [29]. Participants will not be given quantitative dietary advice but will be provided with knowledge about healthy and unhealthy food groups to assist them to make an informed decision on optimal food choice.

#### Physical activity

The physical activity component will follow the same guidelines set out for the participants in the TAKE 5 intervention. However, participants will not be encouraged to self-monitor or increase their daily step counts using a pedometer.

#### Behaviour change techniques

The behaviour change technique goal setting and self-monitoring will be incorporated into the intervention purely with respect to dietary targets and physical activity. Participants will be invited to set a general dietary or a physical activity goal for each session and to self-monitor their food intake and physical activity.

#### Phase 2—weight maintenance

At each session, the dietitian will weigh the participant and discuss their weight in the context of sustaining any weight change. The opportunity for participants or carers to ask any questions relevant to diet and physical activity will be available at every session.

### Outcome measures

A researcher (LH) who is blind to study group allocation is responsible for collecting all outcome measures. Demographic, health questionnaires and all other outcome measures will be completed at baseline, at 6 months and at 12 months.

#### Primary outcome

The primary outcome measure will be the mean difference in body weight (kilograms (kg)) at 12 months from baseline between the two treatment groups.

#### Secondary outcome

Secondary outcomes include weight loss of 5% or more of initial body weight, change in BMI, waist circumference and percentage body fat. Mean percentage time per day spent engaged in moderate-vigorous intensity physical activity, light intensity physical activity and engagement in sedentary behaviour.

#### Anthropometric outcomes

Participants will be invited to have their weight, height, waist circumference and triceps skinfold thickness measured. Measurements will be made with the participant wearing light clothes without shoes. All measurements will be made in duplicate and the final value calculated as the mean of the two measurements. Weight in kilograms will be measured to the nearest 100 grams (g), using SECA877 scales (SE approval class III; SEA Germany). Height in metres (m) will be measured to the nearest 1 mm using the SECA Leicester stadiometer (SECA, Germany). The height (m) and weight (kg) will be used to calculate BMI using the formula BMI = weight/height (kg/m^2^).

Waist circumference will be measured to the nearest 0.5 cm at the midpoint between the iliac crest and the lowest rib, in full expiration whilst the participant is standing [34].

Due to the invasive nature of skinfold measurements and the issue of level of compliance of adults with intellectual disabilities, percentage body fat will be calculated using only the triceps skinfold thickness (mm) measured to the nearest 1 mm, waist circumference (cm) and age (years) of the participant. Separate regression equations for male and female participants, developed by Lean et al. [35] will be used to predict body density and percentage body fat.

#### Physical activity outcome

To objectively measure physical activity, all participants will be invited to wear accelerometers for 7 days on three occasions, at baseline, 6 and 12 months. Accelerometers have been used in studies involving adults with intellectual disabilities and shown to be well utilised by this group and to be a reliable and effective measure of levels of physical activity [36, 37].

Actigraph GT3X+ accelerometers (Manufacturing Technology Inc., Florida) will be worn at the hip, attached to a belt worn round the waist. Participants will be invited to wear the actigraph GT3X+ over a 7-day period. Instructions will be given to wear the actigraph during all waking hours, except when showering, bathing or swimming.

In keeping with guidelines on the validity of accelerometer data, the minimum data requirement will be set at 6 h of data, on at least 3 days from seven [38]. If this requirement is not met, the accelerometer data will be discarded and not be included in the analysis. The number of participants who fail to record sufficient records of physical activity will be recorded. The accelerometers will be set to record activity over 15-s intervals (epochs), with activity counts of four consecutive epochs summed to give activity counts per minute (cpm). Based on recommendations from previous studies [39], four categories of activity intensity will be defined:

- Sedentary behaviour 0–499 cpm.
- Light intensity activity 500–1,951 cpm.
- Moderate intensity activity 1,952–5,724 cpm.
- Vigorous intensity activity greater than 5,725 cpm.

The accelerometer data will then be used to calculate the mean time (minutes) and the percentage time per day, spent in each level of activity.

In addition, physical activity levels will also be measured subjectively by administering the International Physical Activity Questionnaire-Short (IPAQ-S). This will provide information about the types of physical activity they participated in.

### Health-related quality of life

To allow comparison with weight loss studies that do not include adults with intellectual disabilities as participants, the EQ-5D [40] will be used to measure health-related quality of life. The EQ-5D has been shown to be reliable, valid and sensitive to change in adults with obesity [41].

Completion of generic measures, like the EQ-5D, can be difficult for adults with intellectual disabilities because of the levels of communication and abstraction required. The EQ-5D has been used as a proxy-measure of health-related quality of life in studies involving adults with cognitive impairments due to dementia [42, 43] and stroke [44]. In this study, carers will be asked to complete the EQ-5D as a rating of the carer views of the five domains in the EQ-5D, rather than as proxy-rating. Individuals with mild intellectual disabilities will also be asked to complete the EQ-5D and the level of agreement with carer ratings examined. The wording from the youth version of the EQ-5D will be used as it is aimed at young people aged 7 years and older and is appropriate to the of verbal comprehension of adults with mild/moderate intellectual disabilities.

### Process measures

An in-depth process evaluation of the interventions will be conducted as a separate study on completion of the study. Process measures will be collected as part of the treatment protocol, as the research dietitian will also complete written clinical notes at the end of each intervention session, noting the success of components of the interventions, and ways they adapted the approach (in accordance with the manual) to individual need and circumstances. The research dietitian will also collect routine data on the number of sessions attended, participants’ body weight and information about the participants’ dietary habits, physical activity and their success in using the behaviour change techniques. Analysis of process evaluation data will provide insight into multiple aspects of the interventions and will capture experiences gained with delivering the interventions and the fidelity of the interventions which will be used to help inform the development of a full-scale clinical trial.

### Data analysis

The main analysis of the pilot study will include descriptive statistics of feasibility outcomes, including recruitment rates and the acceptability of randomisation (recorded from attrition rates). This analysis will help inform the development of a full-scale trial. Additional analysis of patient-centred outcomes will be carried out according to a detailed pre-specified statistical analysis plan. The primary efficacy analysis, change in body weight at the end of the intervention period (≈12 months) from baseline will use a linear regression (random effects) model to take into account clustering and will be adjusted for randomised group, baseline weight and variables used in the minimisation algorithm (level of intellectual disabilities and presence of Down Syndrome). Similar linear regression models will be fitted for each continuous secondary outcome measure. A logistic regression model will be fitted for the categorical outcome, weight loss of 5% or more of initial body weight taking account of clustering and baseline adjustments listed above. Further analyses may assess the effects of baseline characteristics on outcomes and investigate the evidence for interactions with treatment effects using regression models.

Analyses will be conducted according to the intention to treat (ITT) principle. Per-protocol analyses, including only those participants who engaged with the programme, will also be used to test the sensitivity of the ITT results. Analyses will initially be performed using only those individuals for whom outcome data are available. Participants, who are lost to follow-up, will be assessed to see if they were different at baseline to those included in these analyses. As this is a pilot trial, the level of missing data will be recorded, but no value imputation for outcome variables will be conducted.

## Discussion

The increase prevalence of obesity and health inequalities in adults with intellectual disabilities is further accentuated by the limited access to evidence-based health care in meeting the equality legislation and addressing the health needs of this population group [45]. The need for research on the management of obesity for adults with intellectual disabilities has been emphasised as a priority for research and an important step towards addressing this issue [12, 13].

This pilot study will examine the feasibility of conducting a full-scale trial. The trial will help provide valuable insight into the feasibility of key issues such as the acceptability of randomisation and the fidelity of the intervention delivery.

Both arms of this trial are active interventions designed to support adults to make healthier lifestyle choices, with particularly focussed on supporting weight loss. This study will compare the two dietary strategies, an individualised EDD with quantitative dietary advice against a health education approach without quantitative dietary advice. Both interventions include support to increase physical activity and incorporate behaviour change techniques. Thus, comparing the two interventions will allow the identification of the essential and any superfluous components of the intervention which will be used to inform the optimal management approach to take forward in designing a full-scale clinical trial to address the obesity epidemic in adults with intellectual disabilities.

This study will make important contributions to the available evidence in this field, by piloting an intervention that fully satisfies UK clinical obesity guidelines on weight management. In a recent review of weight management interventions for adults with intellectual disabilities and obesity, it was reported that there are relatively few published studies and that the evidence available is subject to methodological limitations including recruiting small samples sizes with inadequate statistical power, and with only a few studies implementing a randomised controlled design [11]. None of the interventions met current recommendations from UK clinical guidelines on the use of multi-component interventions with an EDD for weight loss [12, 13]. Studies instead include combinations of a component relevant to supporting individuals to increase levels of physical activity, a dietary advice/health education components or a component with a focus on behavioural change. Furthermore, only a few studies were able to demonstrate a clinically significant weight loss of 5%–10% of initial body weight and met the recommended minimum follow-up period of 12 months to examine the effectiveness of a weight loss intervention.

Current guidelines on weight management interventions recommend weight maintenance as an integral component, illustrating that individuals who have lost a clinically significant weight loss and are able to maintain this weight have made substantial lifestyle changes that will prevent future weight gain or health risks [12, 13]. However, research for weight management in the general population and adults with intellectual disabilities has mainly focused on the development and evaluation of weight loss strategies and has not examined extensively the effectiveness of weight maintenance interventions that immediately follow a weight loss phase [46]. Only four studies involving participants with intellectual disabilities offered a structured weight loss maintenance intervention [24, 25, 47, 48]. Of these, only one study, reported the long-term effects of a 6-month weight maintenance intervention [45], illustrating that after 6 months, most adults with intellectual disabilities were able to sustain the clinically significant weight lost in phase 1 or continue further to lose weight. This study will therefore add to the evidence base by piloting a structured weight maintenance phase.

The controlled design is a key strength of this study, as it is considered the optimal design to evaluate complex interventions [49] and will add to the limited evidence of controlled studies of weight management interventions of adults with intellectual disabilities and obesity. There is limited evidence on which to base a best alternative control intervention. An ‘active’ control intervention is selected for this study over a non-intervention control; as in addition to the ethical issues discussed previously, the research group believes that this design would threaten the validity of the study by not taking into consideration the specific treatment effects of the intervention. In addition, it is believed that offering participants no intervention would have a negative effect on study recruitment and retention of participants. Receiving information on the importance of weight management may influence participants receiving a non-intervention control to change their behaviour and seek out other resources available in order to make healthier lifestyle choices. This could potentially cause individuals to drop out from the study and/or make it difficult to follow individuals up when offering them no intervention. The findings of this study will provide clear direction on this key issue and guide future experimental designs.

It is recognised that specific challenges are likely to arise when conducting research with adults with intellectual disabilities due to their cognitive and communication needs. In particular, it is noted that recruitment of adults with intellectual disabilities to research studies is challenging and is evident from the small sample sizes reported in previous research [11]. A review by Cleaver et al. [50] reported that ethical procedures which are consistent with this study such as the inability to contact potential participants directly and the procedures of taking consent may lead to poor participation in studies involving adults with intellectual disabilities. This study will assess the feasibility of recruiting and retaining adults with intellectual disabilities to a randomised design of a weight management intervention using a multi-point recruitment strategy which will collect information from recruitment sites such as organisations and services for adults with intellectual disabilities. This will be used to identify potential barriers and facilitators to recruitment which will help inform other controlled trials of weight management interventions and also be influential in informing the development a full-scale multi-centre clinical trial.

## Conclusion

There is limited access to evidence-based health care for adults with intellectual disabilities. Currently, no published controlled studies of weight management interventions for adults with intellectual disabilities meet clinical obesity recommendations. This pilot randomised trial will examine the acceptability of randomisation and attrition rates and provide pilot data on the estimates of patient-centred outcomes of TAKE 5 compared to the health education control intervention. If this study design is acceptable to adults with intellectual disabilities and shows a significant effect on outcome measures, this protocol can serve as a framework or model on which development of a full-scale clinical trial can be based.

## Trial status

Recruitment commenced in February 2014 and is projected to be complete by November 2014.

## Abbreviations

BMR: Basal metabolic rate
BMI: Body mass index
cpm: Counts per minute
EDD: Energy deficit diet
FSA: Food Standards Agency
GCWMS: Glasgow & Clyde Weight Management Service
ITT: Intention to treat
IVRS: Interactive voice response system
IPAQ-S: International Physical Activity Questionnaire-Short version
NHS: National Health Service
MRC: Medical Research Council
WWToo: Waist Winners Too
PARQ: Physical Activity Readiness Questionnaire
TAU: Treatment as usual.

## References

[1] *American Association on Intellectual and Developmental Disabilities: Definition of Intellectual Disability*. [http://aaidd.org/intellectual-disability/definition]

[2] Hove O (2004). Weight survey on adult persons with mental retardation living in the community. Res Dev Disabil.

[3] Emerson E (2005). Underweight, obesity and exercise among adults with intellectual disabilities in supported accommodation in Northern England. J Intellect Disabil Res.

[4] Yamaki K (2005). Body weight status among adults with intellectual disability in the community. Ment Retard.

[5] Melville CA, Cooper SA, Morrison J, Allan L, Smiley E, Williamson A (2008). The prevalence and determinants of obesity in adults with intellectual disabilities. J Appl Res Intellect Disabil.

[6] Bhaumik S, Watson JM, Thorp CF, Tyrer F, McGrother CW (2008). Body mass index in adults with intellectual disability: distribution, associations and service implications: a population-based prevalence study. J Intellect Disabil Res.

[7] Wilson PW, D’Agostino RB, Sullivan L, Parise H, Kannel WB (2002). Overweight and obesity as determinants of cardiovascular risk: the Framingham experience. Arch Int Med.

[8] Hu G, Tuomilehto J, Silventoinen K, Barengo NC, Peltonen M, Jousilahti P (2005). The effects of physical activity and body mass index on cardiovascular, cancer and all-cause mortality among 47 212 middle-aged Finnish men and women. Int J Obes.

[9] Hu G, Lindstrom J, Valle TT, Eriksson JG, Jousilahti P, Silventoinen K (2004). Physical activity, body mass index, and risk of type 2 diabetes in patients with normal or impaired glucose regulation. Arch Int Med.

[10] Cooper SA, Melville C, Morrison J (2004). People with intellectual disabilities. Their health needs differ and need to be recognised and met. Br Med J.

[11] Spanos D, Melville CA, Hankey CR (2013). Weight management interventions in adults with intellectual disabilities and obesity: a systematic review of the evidence. Nutr J.

[12] National Institute for Health and Clinical Excellence (NICE) (2006). Obesity: The Prevention, Identification, Assessment and Management of Overweight and Obesity in Adults and Children.

[13] Scottish Intercollegiate Guideline Network (SIGN) (2012). Management of Obesity: A National Clinical Guideline.

[14] Morrison DS, Boyle S, Morrison C, Allardice G, Greenlaw N, Forde L (2011). Evaluation of the first phase of a specialist weight management programme in the UK National Health Service: prospective cohort study. Pub Health Nutr.

[15] Melville CA, Boyle S, Miller S, Macmillan S, Penpraze V, Pert C (2011). An open study of the effectiveness of a multi-component weight-loss intervention for adults with intellectual disabilities and obesity. Br J Nutr.

[16] Spanos D, Hankey CR, Boyle S, Koshy P, Macmillan S, Matthews L (2012). Carers’ perspective of a weight loss intervention for adults with intellectual disabilities and obesity: a qualitative study. J Intellet Disabil Res.

[17] Spanos D, Hankey CR, Melville CA (2013). Comparing the effectiveness of a multi-component weight loss intervention in adults with and without intellectual disabilities. J Human Nutr Diet.

[18] Spanos D (2013). Weight Loss and Weight Maintenance Interventions for Adults with Intellectual Disabilities.

[19] Guh DP, Zhang W, Bansback N, Amarsi Z, Birmingham CL, Anis AH (2009). The incidence of co-morbidities related to obesity and overweight: a systematic review and meta-analysis. BMC Public Health.

[20] Whitlock G, Lewington S, Sherliker P, Clarke R, Emberson J, Halsey J (2009). Body-mass index and cause-specific mortality in 900 000 adults: collaborative analyses of 57 prospective studies. Lancet.

[21] Thomas S, Reading J, Shephard R (1992). Revision of the physical activity readiness questionnaire (PAR-Q). Can J Sports Sci.

[22] Cooper SA (1997). Epidemiology of psychiatric disorders in elderly compared with younger adults with learning disabilities. Br J Psychiat J Ment Sci.

[23] Sparrow SS, Balla DA, Cicchetti DV (1984). Vineland Adaptive Behavior Scales.

[24] Fox RA, Haniotes H, Rotatori A (1984). A streamlined weight loss program for moderately retarded adults in a sheltered workshop setting. Appl Res Ment Retard.

[25] Fox RA, Rosenberg R, Rotatori AF (1985). Parent involvement in a treatment program for obese retarded adults. J Behav Ther Exp Psychiatry.

[26] Mifflin MD, St Jeor ST, Hill LA, Daugherty SA, Koh YO (1990). A new predictive equation for resting energy expenditure in healthy individuals. Am J Clin Nutr.

[27] Leslie WS, Lean ME, Baillie HM, Hankey CR (2002). Weight management: a comparison of existing dietary approaches in a work-site setting. Int J Obes Rel Metabol Disord.

[28] Murphy J, Cameron L (2008). The effectiveness of Talking Mats® with people with intellectual disability. Brit J Learn Disabil.

[29] Food Standards Agency (2009). The Eatwell Plate.

[30] Department of Health (2004). At Least Five a Week. Evidence for the Impact of Physical Activity and its Relationship to Health.

[31] NHS Health Scotland (2009). Five Year Review of “Let’s Make Scotland More Active” - A Strategy for Physical Activity.

[32] O'Donovan G, Blazevich AJ, Boreham C, Cooper AR, Crank H, Ekelund U (2010). The ABC of physical activity for health: a consensus statement from the British association of sport and exercise sciences. J Sports Sci.

[33] National Institute for Health and Clinical Excellence (NICE) (2007). Behaviour Change at Population, Community and Individual Levels.

[34] World Health Organisation (2008). WHO STEPwise Approach to Surveillance (STEPS).

[35] Lean ME, Han TS, Deurenberg P (1996). Predicting body composition by densitometry from simple anthropometric measurements. Amer J Clin Nutr.

[36] Temple VA, Walkley JW (2000). Physical activity of adults with intellectual disability. J Intellect Develop Disabil.

[37] Temple VA, Walkley JW (2007). Perspectives of constraining and enabling factors for health promoting physical activity. J Intellet Develop Disabil.

[38] Penpraze V, Reilly JJ, MacLean C, Montgomery C, Kelly L, Paton JY (2006). Monitoring of physical activity in young children: how much is enough?. Pediatr Exer Sci.

[39] Freedson PS, Melanson E, Sirard J (1998). Calibration of the computer science and applications, Inc. Accelerometer. Med Sci Sports Exer.

[40] Brooks R (1996). EuroQol: the current state of play. Health Pol.

[41] Sach TH, Barton GR, Doherty M, Muir KR, Jenkinson C, Avery AJ (2007). The relationship between body mass index and health-related quality of life: comparing the EQ-5D, EuroQol VAS and SF-6D. Int J Obes.

[42] Coucill W, Bryan S, Bentham P, Buckley A, Laight A (2001). EQ-5D in patients with dementia: an investigation of inter-rater agreement. Med Care.

[43] Jonsson L, Andreasen N, Kilander L, Soininen H, Waldemar G, Nygaard H (2006). Patient- and proxy-reported utility in Alzheimer disease using the EuroQoL. Alz Dis Assoc Dis J.

[44] Pickard AS, Johnson JA, Feeny DH, Shuaib A, Carriere KC, Nasser AM (2004). Agreement between patient and proxy assessments of health-related quality of life after stroke using the EQ-5D and Health Utilities Index. Stroke.

[45] Michael J (2008). Healthcare for All: Report of The Independent Inquiry Into Access to Healthcare for People with Learning Disabilities.

[46] Avenell A, Broom J, Brown TJ, Poobalan A, Aucott L, Stearns SC (2004). Systematic review of the long-term effects and economic consequences of treatments for obesity and implications for health improvement. Health Technol Assess.

[47] McCarran MS, Andrasik F (1990). Behavioral weight-loss for multiply-handicapped 4 adults: assessing caretaker involvement and measures of behavior change. Addict Behav.

[48] Saunders RR, Saunders MD, Donnelly JE, Smith BK, Sullivan DK, Guilford B (2011). Evaluation of an approach to weight loss in adults with intellectual or developmental disabilities. Intellect Dev Disabil.

[49] MRC (2008). Developing and Evaluating Complex Interventions: New Guidance.

[50] Cleaver S, Ouellette-Kuntz H, Sakar A (2010). Participation in intellectual disability research: a review of 20 years of studies. J Intellect Disabil Res.

